# Right dorsal colon ultrasonography in normal adult ponies and miniature horses

**DOI:** 10.1371/journal.pone.0186825

**Published:** 2017-10-24

**Authors:** Natalia Siwinska, Agnieszka Zak, Monika Baron, Marta Cylna, Hieronim Borowicz

**Affiliations:** 1 Department of Internal Medicine and Clinic of Diseases of Horses, Dogs and Cats, Faculty of Veterinary Medicine, University of Environmental and Life Sciences, Wroclaw, Poland; 2 Faculty of Veterinary Medicine, University of Environmental and Life Sciences, Wroclaw, Poland; Massewy University, NEW ZEALAND

## Abstract

The aim of this study was to determine the normal location, wall thickness and motility of the right dorsal colon in adult ponies and miniature horses. The abdominal ultrasonography examination was performed in a study group consisting of 23 ponies and miniature horses and in a control group comprising ten Thoroughbred horses. The procedure was performed in unsedated standing animals. The location and the thickness of the right dorsal colonic wall was examined on the right side of the abdomen between the 10^th^ and the 14^th^ intercostal space. The contractility was recorded in the 12^th^ intercostal space. A comparative analysis between the study group and control group was carried out using the Student’s t-test. Pearson’s linear correlation coefficient was used to calculate the correlation between the thickness of the colonic wall as well as the number of peristaltic movements and age, wither height and body mass of the animals. The right dorsal colon was identified in all the horses in the 12^th^ intercostal space. In all the intercostal spaces the mean ± standard deviation (SD) wall thickness of the right dorsal colon was 0.27 ± 0.03 cm in the horses from the study group and 0.37 ± 0.03 cm in the control horses. The mean number of peristaltic contractions was 4.05 ± 1.07 per minute in the animals from the study group and 1.7 ± 0.46 contractions per minute in the control group. The values of the ultrasonographic wall thickness and peristaltic motility in small breed horses in the present study were different from the values obtained for large breed horses. The study also found that the right dorsal colon in small breed horses is physiologically located in the 12^th^ intercostal space. This suggests that different reference values should be used in small horse breeds when performing an ultrasound examination.

## Introduction

The equine large intestine is a highly specialized section of the digestive tract responsible for fibre digestion [1,2]. The function, anatomic structure and ability to move within the abdominal cavity, predispose the large intestine to conditions that cause colic such as impaction, displacement, distention, torsion, sand accumulation [2–7]. A retrospective analysis of 151 horses referred for a laparotomy showed that 53% had lesions in the large intestine [8,9]. Recent data presented by Curtis *et al*. carried out on 1016 horses revealed that large colon impaction and simple displacement are the second most common cause of colic [7]. It is difficult to conduct an accurate examination of the digestive tract in horses due to the large size of their abdominal cavity. While 30–40% of the peritoneal cavity can be examined via a rectal examination, it does not allow assessment of the intestine function and thickness [10]. In horses, the percutaneous abdominal ultrasound examination is useful and complements the rectal examination in animals with intestinal disease [11–17]. The method is non-invasive, fast, does not require highly-specialized equipment and does not cause any side-effects. Furthermore, it facilitates the abdominal cavity examination of non-cooperative animals in which a rectal examination cannot be performed [13,17]. The ultrasound examination reliability has been confirmed by several authors [18–23].

Thickening of the intestinal wall may occur due to inflammation, with or without inflammatory cell infiltration (e.g. inflammatory bowel disease—IBD), neoplastic cell infiltration (e.g. *lymphoma*) or oedema caused by venous congestion triggered by an occlusion (volvulus) [18–22]. In horses, inflammation of the right dorsal colon (RDC) can be associated with non-steroidal anti-inflammatory drug administration and affects a single segment of the intestines [24]. Thickening of the intestinal wall also causes narrowing of the lumen, leading to obstruction, indigestion and malabsorption, which in turn causes recurring colic, chronic diarrhoea or progressive weight loss [21].

To date, the only reference values of the thickness and peristaltic movements of the equine colon have been provided for large breed horses and foals [25,26,27]. The digestive tract of healthy, unpremedicated ponies has been evaluated using ultrasound imaging and included the assessment of the stomach, duodenum, jejunum and cecum [28]. There is no available data on the thickness of the large intestinal wall and its motility in miniature breed horses and ponies. In other animals, a species-specific relationship was demonstrated between body weight, animal size and thickness of the colonic wall [29]. It is not known whether this variation occurs in horses, and determining normal ranges and variations in the thickness of the intestinal wall and the number of peristaltic contractions is important for interpretation of clinical findings.

The aim of the study was to determine the normal reference ranges for ultrasound parameters of the RDC in ponies and miniature breed horses. The three objectives of the study were to determine the physiological parameters: (1) the location of ultrasonographic visualization of the RDC, (2) the mean wall thickness in all intercostal spaces occupied by the RDC, (3) the mean number of peristaltic movements in this section of the gastrointestinal tract in small breed horses.

## Materials and methods

### Animals

The study was carried out on 33 horses. The study group consisted of 23 small breed horses– 13 ponies and ten miniature breed horses—ranging from four to 22 years of age (mean ± standard deviation [SD] 11.8 ± 4.8 years), of various breeds (eight Shetland ponies, two Welsh ponies, one Fjord horse, one Felinski pony, one Polish pony and ten crossbreds). There were 13 females and ten males, with a wither height ranging from 70 to 130 cm (99.6 ± 17.2 cm). The body condition score of each horse was assessed at 5–6 points on a nine point scale [30]. The animals weighed from 118 to 380 kg (223.1 ± 73.5 kg). The control group chosen for the ultrasound examination included ten adult Thoroughbreds (five males and five females) from five to seven years old (5.4 ± 0.7 years). In that group, the mean body weight ranged from 498 to 574 kg (508.1 ± 31.7 kg), the wither height ranged from 154 to 168 cm (159.5 ± 4.1 cm) and the body condition score of each horse was assessed at five point on a nine point scale [30]. The animals were included in the study based on a complete history provided by their owners (primarily no history of gastrointestinal disease and treatment within the previous 12 months) and following a clinical examination, which mainly assessed the behavior (regular appetence), rectal temperature, intestinal sounds, stool formation and exclusion of sand in faeces. The assessed parameters were normal in all animals and there were no visible clinical signs of any disease. There were no changes in the stable environment of each horse three years prior to the study and no dietary changes for at least a year prior to the examination. Each animal was vaccinated regularly and dewormed according to an established anthelmintic program determined for the entire team of horses. The last deworming (using ivermectin) was carried out seven weeks prior to the study. None of the mares was pregnant. Depending on their body weight, the animals were fed oats twice a day (6 a.m. and 6 p.m.) and had unlimited access to hay and drinking water. The horses were kept in a stable-pasture system with a daily 5-hour access to green pasture. The animals were not sedated for the study. They had different owners but resided in the same private horse riding center in Lower Silesia, Poland. The owners consented to the study and the publication of its results.

### Ultrasound examination

**Fig 1:**
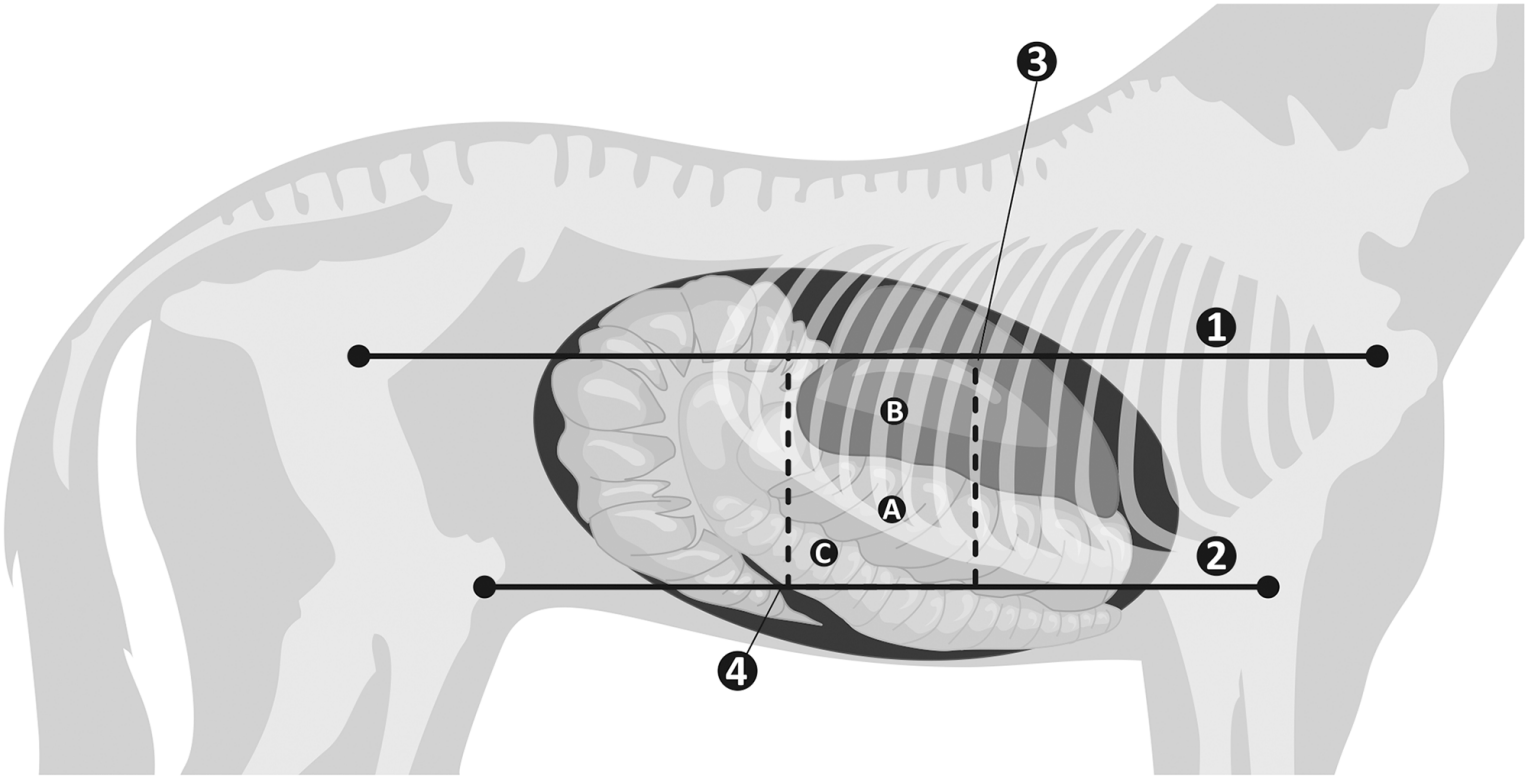
**Schematic illustration of the right side of the abdomen for the examination of the right dorsal colon—RDC (A).** The imaging window was allotted using four lines: line 1 –from the shoulder joint to the coxal tuberosity, line 2 (parallel to line 1)–from the elbow joint to the stifle joint, line 3 –a line along the 9^th^ intercostal space, line 4 (parallel to line 3)–a line along the 15^th^ intercostal space. The liver was visible next to the RDC (B), the right ventral colon was visible below the RDC (C).

The ultrasound examinations were performed on all the horses in the stable corridors (between 1 and 3 p.m.). The right side of the abdominal wall was shaved from the 9^th^ to the 15^th^ intercostal space (ICS), at the level of a line drawn from the point of the shoulder joint to the coxal tuberosity and from the elbow joint to the stifle joint (Fig 1) using an electric clipper with blade No. 40.

The ultrasound examination was carried out using a MyLab ^™^ 30Gold VET scanner equipped with a multifrequency (1–4 MHz) convex transducer (Esoate). The site was washed with chlorhexidine-based soap and warm water in order to ensure direct contact between the transducer and the skin. The site was then washed with alcohol, and the ultrasound gel was applied to the skin. The ultrasound examination was carried out in a prepared diagnostic window, as reported in the literature, also known as the duodenal window in the FLASH (fast localized abdominal sonography of horses) examination [31,32,33]. The examination was always initiated in the 9^th^ ICS and carried out caudally, until the RDC was no longer visible. The transducer was moved in a dorsal to ventral direction in order to visualize the intestinal wall. The depth of the ultrasound beam penetration was adjusted to maximize image quality in individual subjects. The RDC was identified based on its appearance (formation of semicircles, caused by bowel gas reflections of ultrasound beams), size (large diameter) and location [14,31]. The wall of the RDC was identified by its location, with axial and ventral proximity to the liver, ventral proximity to the duodenum and dorsal proximity to the right ventral colon [21]. The dorsal colon was differentiated from the ventral colon based on the presence of a non-sacculated wall [14,21,31]. In all the studied horses, the location of the RDC was assessed in the ultrasound examination based on the ICS it occupied in the diagnostic window. The colonic wall thickness was assessed from the 9^th^ to the 15^th^ ICS. The wall of the colon was hypoechogenic or echogenic, with hyperechogenic gas reflecting the ultrasound beams from the surface of the mucosa, thus obstructing visualization of the bowel content [32]. The thickness of the wall was measured in centimeters from the hyperechoic zone of the serosa to the hyperechoic zone of the submucosa using a software-based electronic calliper. All the obtained images were recorded and stored as soft copies (DICOM images) for further analysis. The measurements were taken three times in each ICS. Two hundred and sixty-seven measurements were collected in the study group and 150 measurements were obtained from the control group, giving 417 measurements of the colonic wall. The mean value for each ICS measurements was calculated. In all horses, the RDC was visualized at the 12^th^ ICS level. Hence, it was retrospectively selected as the measurement site of the peristaltic movements per minute. The peristaltic contraction was recognized as a centripetal movement of the intestinal wall causing a decrease in the intestinal lumen size [2]. Peristaltic contraction type and direction were not assessed. All the measurements were carried out by one researcher (NS), who holds a license to perform studies on animals (number 105/2016). The number of peristaltic contractions was simultaneously assessed by four researchers (NS, AZ, MB, MC). All of the examinations performed in this study were non-invasive and are routinely performed in everyday medical practice. In accordance with the existing law applicable in Poland, Experiments on Animals Act from January 15^th^ 2015 (Journal of Laws of the Republic of Poland, 2015, item. 266), non-invasive clinical studies do not require ethical approval.

### Statistical analysis

Descriptive statistics were used to assess the location of the colon. The continuous and discrete data are presented as mean ± standard deviation (SD). The mural thickness and the number of peristaltic contractions of the RDC were compared between the study and control groups. In the study group, the mean wall thickness and peristaltic contractions of the colon were compared between the genders. Due to the fact that the data were normally distributed, comparative analyses were carried out using the Student’ t-test for independent samples. In the study group, parameter estimation in linear regression analysis and the Pearson correlation coefficient as well as the subsequent significance were used to assess the relationship between the age, wither height, body weight and the mean thickness of the dorsal colon wall in all the ICS and also the number of peristaltic movements per minute. The significance level was set at 5% and all the calculations were carried out using the StatSoft, Inc Statistica for Windows software.

## Results

**Fig 2:**
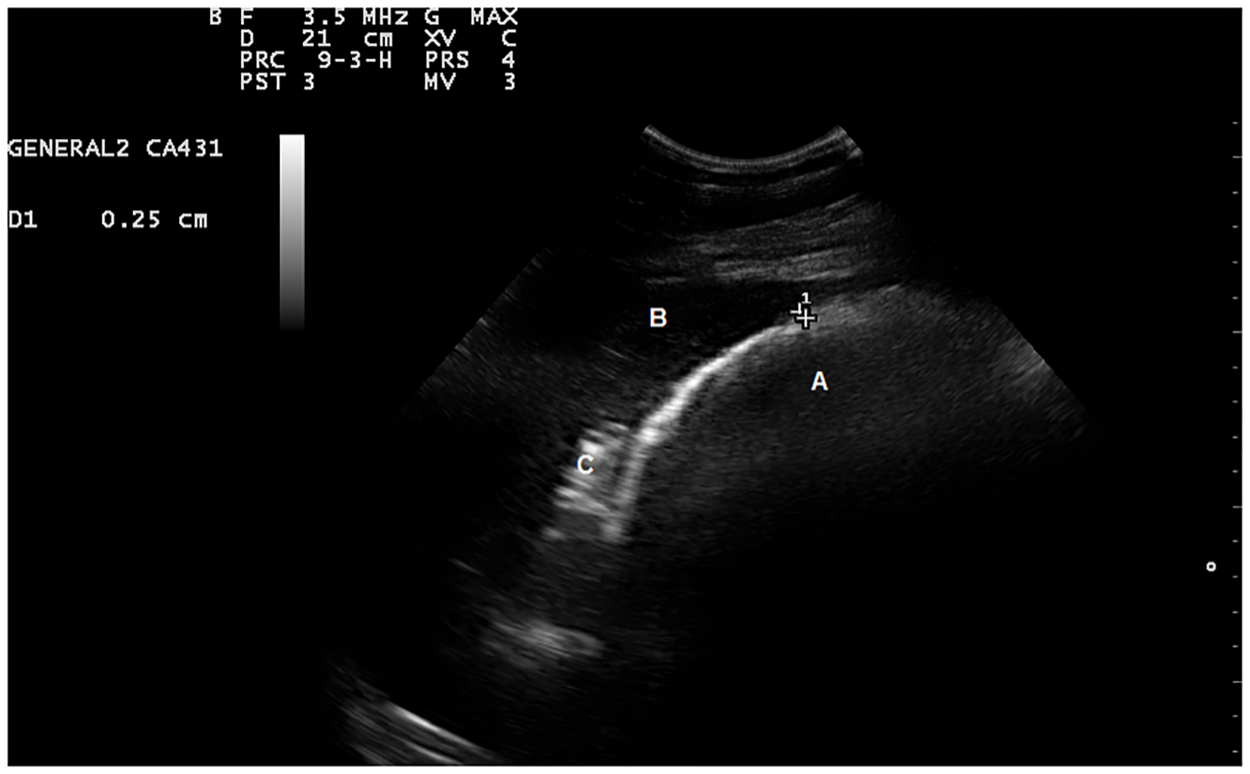
A normal sonogram of the right dorsal colon in a healthy pony. Image obtained by placing a convex transducer in the right 12^th^ intercostal space. The right dorsal colon (A) was identified as a semicircle with bowel gas beam reflections. The liver (B) is seen in the upper hand corner of the image, the duodenum (C) is seen between the liver and the right dorsal colon. The measured thickness of the colonic wall was 0.25 cm.

The complete RDC assessment was tolerated well by all the animals and lasted from five to eight minutes per horse, depending on the degree of intestinal wall visualization. The RDC was visible and identified in all the animals in both groups. There were no abnormalities in the ultrasound appearance of the RDC in any of the animals. The image formed semicircles, which were caused by the bowel gas reflection of ultrasound beams (Fig 2).

The content of the colon was normal (visible gas). In all the animals, the RDC was physiologically located as described in literature. The anatomic site of the RDC extended across five ICS although the exact range differed between individuals (Table 1).

**Table 1:** The percentage of organ visibility in different intercostal spaces (Examination window right dorsal colon) in 23 examined ponies and miniature horses.

Region	Lungs	Right Dorsal Colon	Right Ventral Colon
10^th^ ICS	43%	57%	-
11^th^ ICS	13%	87%	-
12^th^ ICS	-	100%	-
13^th^ ICS	-	96%	4%
14^th^ ICS	-	70%	30%

*Note*: ICS—intercostal space

The dorsal colon was not identified in the 9^th^ ICS as the image was obscured by the lungs in all the animals. In some animals from the study group, the RDC was also covered by the lungs in the 10^th^ and 11^th^ ICS. In a small percentage of animals from the study group, it was not visible in the 13^th^ and 14^th^ ICS. Similarly, the dorsal colon was not visible in the 15^th^ ICS in all the animals. In the ventral part of the abdomen, the RDC passed into the right ventral colon, which contained clear sacculation. The image of the dorsal colon was obtained in five consecutive ICS (10^th^-14^th^) in the whole control group but only in seven horses from the study group (30.4%). The RDC was identified in all horses in the 12^th^ ICS. No relationship was observed between the location of the colon and age, sex or body weight of the animals.

### The thickness of the intestinal wall

The echogenicity pattern of the RDC in all the horses was similar. The hyperechogenic, hypoechogenic and a hyperechogenic layer with gas reflection were visible in all the animals. The results of the measurements of the mural wall thickness in each ICS in horses from the study and control groups are presented in Table 2.

**Table 2:** Right dorsal colon wall thickness visible using ultrasonography in 23 ponies and miniature horses and its comparison with values obtained from 10 large breed horses (Thoroughbreds).

	Study Group—Ponies and Miniature Horses		Control Group—Large Breed Horses	
Region	Mean Wall Thickness ± SD (cm)	Range (Minimum—Maximum Value) (cm)	Mean Wall Thickness ± SD (cm)	Range (Minimum—Maximum Value) (cm)
10^th^ ICS	0.27 ± 0.03*	0.21–0.31	0.37 ± 0.03	0.35–0.43
11^th^ ICS	0.27 ± 0.02*	0.22–0.32	0.37 ± 0.04	0.34–0.44
12^th^ ICS	0.28 ± 0.02*	0.26–0.32	0.35 ± 0.02	0.33–0.38
13^th^ ICS	0.27 ± 0.02*	0.22–0.30	0.37 ± 0.03	0.34–0.42
14^th^ ICS	0.28 ± 0.02*	0.25–0.32	0.37 ± 0.03	0.35–0.4

*Note*: ICS—intercostal space; SD—standard deviation.

*Statistically significant (*P*<0.001).

The mean thickness of the RDC at all the sites was 0.27 ± 0.03 cm in the study group and 0.37 ± 0.03 cm in control horses. The largest difference in the colonic wall thickness in horses in the study group was 0.12 cm and 0.1 cm in the control group. The maximum difference in the mural thickness between the measurement sites was 0.11 cm in all horses and 0.07 cm in individual.

The mural wall thickness of the RDC in all the ICS was significantly lower (*P*<0.001) in the study group than in the large breed horses from the control group (Table 2). The mean thickness of the RDC wall did not differ (p = 0.248) between males (0.28 ± 0.02 cm) and females (0.27 ± 0.02 cm). In the study group, there was a positive correlation between the wither height and the thickness of the colonic wall (r = 0.49, p = 0.018). There was no correlation between the thickness of the RDC wall and the body mass (r = -0.072, p = 0.746) or age (r = -0.030, p = 0.890).

### Number of peristaltic contractions

The mean number of peristaltic contractions in all the ponies and miniature horses was 4.05 ± 1.07 contractions/minute. The lowest number of peristaltic contractions was two per minute while the highest number was six per minute. In the control group, there were one to two peristaltic contractions, while the mean number of contractions was 1.7 ± 0.46 per minute. The number of peristaltic contractions in the colon in ponies and miniature horses was significantly greater (*P*<0.001) than the values in large breed horses. The mean number of peristaltic contractions did not differ (p = 0.34) between males (4.4 ± 1.7 per minute) and females (3.9 ± 0.9 per minute) in the study group. There was no correlation between the number of peristaltic contractions and the age (r = 0.130, p = 0.555), body weight (r = 0.011; p = 0.960) or wither height (r = 0.264, p = 0.223) of the studied horses.

## Discussion

The ultrasound evaluation of the intestines in horses can be carried out transrectally or transabdominally [9,15]. Numerous studies have shown that percutaneous transabdominal ultrasonography enables accurate evaluation of the location and appearance of a chosen segment of the digestive tract as well as the measurement of its thickness [9,17,20,21]. An ultrasound examination is the only test that allows a non-invasive real-time assessment of peristaltic contractions in horses [1,2,26,27]. It was well tolerated by all the studied animals, since it lasted no longer than several minutes. Moreover, in small breed horses, a transrectal ultrasound examination cannot be performed due to the animal size. Hence, a percutaneous transabdominal ultrasound examination is the method of choice for non-invasive diagnosis of disorders in the abdominal cavity [9].

According to Smith *et al*. [34], the use of a linear or convex transducer to assess the intestinal wall gives an optimum image resolution and increases the replicability of the results. The authors used a convex transducer, which is most commonly used in horses [31]. Due to its low frequency, it provides a larger depth of penetration of the ultrasound wave, enabling its use in the large equine abdominal cavity. However, the low frequency of the transducer may affect the image quality. High-resolution transducers may enable better image quality with enhanced detail of the intestinal wall and more accurate measurements [18]. This, though, would reduce the penetration depth of the ultrasound beam, which, in turn, may negatively affect the assessment of the intestinal wall in animals with a thick layer of subcutaneous fat. In the healthy horse, the intestinal wall is composed of five layers (serosal, muscular, submucosal, mucosal and mucosal-luminal) [9,15]. In the ultrasound examination, it is possible to visualize only three of them: the hyperechogenic mucosal, the hypoechogenic muscular and the hyperechogenic serosal layer. If the wall of the colon is not thickened, it may be difficult to differentiate the luminal edge of the mucosal layer from the contents of the intestine [14,21]. This could cause an overestimation of the thickness of the RDC. In order to reduce the equipment and examination technique bias, the measurements in both groups were collected in the same way, using the same equipment and by the same examiner experienced in performing ultrasound examinations.

In large breed horses, the RDC in the ultrasound examination is located relatively superficially in the right abdominal cavity in the middle 1/3 of the abdominal cavity, from the 10^th^ to the 14^th^ ICS [9,21,24]. This was confirmed by the results obtained in Thoroughbreds, where this location is considered fixed. In ponies and miniature horses, the location was similar although the RDC occupied various ICS and was visible in all five ICS in only 30.4% of the horses. The lungs masked the RDC in more cranial ICS, while the right ventral colon covered the RDC in more caudal ICS. Jones *et al*. carried out an ultrasound examination in horses with right dorsal colitis and found that the RDC was not visible in all five ICS, but was regularly identified in the 11^th^, 12^th^, and 13^th^ right ICS [21]. In the whole study group, the RDC was present in the 12^th^ ICS. This suggests that this particular ICS may be the optimal imaging site of the RDC when performing an ultrasound examination in normal ponies and miniature horses. Other studies have not reported an optimal location to measure the RDC in this type of horse. Galvin *et al*. also chose to examine the RDC wall thickness in large breed horses with right dorsal colitis in the 12^th^ ICS [24]. Hence, it may be assumed that this particular ICS provides the best site for RDC analysis. The identification of a universal anatomical location for the RDC analysis is extremely important, especially during the rapid FLASH examination [31]. The imaging window used to assess the RDC in the FLASH protocol proposed by Busoni *et al*. does not include the 12^th^ ICS [33]. The examination of the RDC may be omitted when using this protocol. The study carried out by Epstein *et al*. also reported a different location of the stomach in ponies compared to large-breed horses [28]. These differences in the ultrasound findings between large horses and ponies/miniature horses should be considered when assessing clinical cases and interpreting potential abnormalities and displacements. The present study shows that a more organ specific approach is needed to create an ultrasound protocol for ponies and miniature horses. In order to determine the best diagnostic location of RDC lesions in small breed horses, future studies should include animals with lesions in this area.

The mean colonic wall thickness in ponies and miniature horses was smaller than that noted in the control group of large breed horses in this study, and in previously reported studies [21]. Epstein *et al*. obtained similar results when assessing differences in wall thickness of the stomach, duodenum and jejunum between ponies and large breed horses [28]. Establishing normal reference ranges and breed variations is essential to interpret wall thickening associated with clinical disease. Such differences may be linked to the animal size as a positive correlation between the wither height and the thickness of the colonic wall was identified. In dogs, a relationship between the animal size and the intestinal wall thickness has also been found, with the intestinal wall thicker in larger dogs [29].

The thickness of the intestinal wall depends on the content of the intestines and the intensity of the peristaltic contractions [18,35,36]. In humans, the degree of intestinal filling leads to a decrease in the thickness of the intestinal wall by up to 2 mm [35]. The study by Norman *et al*. found that subjecting healthy horses to a 24 hours fast led to an improved ultrasound image of the small intestine by reducing the amount of ingesta and gas in the large colon [37]. Conversely, there are no such studies assessing the imaging of the equine large intestine. According to previous reports in humans, it may be assumed that decreasing the ingesta in the intestines would lead to a thickening of the intestinal wall [35]. Subjecting horses to long fasts is often impossible and not advisable from a practical and clinical point of view. In the current study, the ultrasonography examination was carried out in all the horses between fodder feedings. The type of diet may also affect the thickness of the intestinal wall [38]. In order to limit the impact of these factors on the results, all the horses in this study were fed a uniform diet at the same feeding times for at least one year preceding the study. Some differences in the thickness of the colonic wall were noted between horses in the same groups, which may be attributed to various diet fed to growing horses. However, there are no studies confirming this supposition.

The mean colon contractility in the study group was higher than reported previously in large breed horses, which corresponded to the results obtained in the control group in the current study [17,39]. The results of Gomaa *et al*., who reported 5.1 ± 0.3 contractions per 3 minutes in the left colon in large breed horses, are similar to those obtained in the control group but also significantly lower than in the study group [1]. Mitchell *et al*. also reported fewer peristaltic movements compared to those in the present study [26]. Similarly, the number of peristaltic contractions in the large intestine reported by Williams *et al*. was significantly smaller (0.8 ± 0.3 per minute) than our findings in both groups [2]. However, those studies did not specifically assess the contractility of the RDC. Williams *et al*. revealed significant differences in the number of peristaltic contractions in different parts of the large intestine [2]. Hence, it is difficult to compare the results of the present study with those provided in literature.

Several factors, such as diet, animal maintenance and time of day may affect intestinal peristalsis [2,26,40,41]. Previous studies reported a decrease in the intestinal motility in fasting or sedated animals. This phenomenon was observed mostly in the small intestine [26]. In the study by Norman *et al*., slight acceleration of jejunal motility was observed after an eight hour fast [37]. Mitchell *et al*. observed decreased intestinal motility in fasted horses [26]. However, colonic activity was not significantly affected, so it may be less sensitive to this factor. In the present study, all the measurements were collected from fed and unsedated horses. The type of maintenance may affect colonic motility. Williams *et al*. found that horses that grazed on fresh pasture had more peristaltic contractions than horses fed and kept in stables [2]. The number of peristaltic movements in grazing horses is similar to those obtained in the control group in the present study, and it is significantly lower than the number obtained in the studied ponies [2]. All the animals in the present study were kept in uniform conditions in a stable-pasture system. It has also been shown that the peristaltic contractile activity in horses depends on the time of day and increases in the afternoon [2]. In order to limit the effect of the time of day on motility fluctuations, all the animals were examined at the same time, between meals.

An increased number of colonic contractions in the studied ponies and miniature horses may be a normal finding associated with the size of the animal, as is the case of the intestinal wall thickness. Similar findings were reported by Epstein *et al*., who found significantly more peristaltic movements in the duodenum of healthy ponies than in large breed horses [28]. Williams *et al*. suspected that the horse size may affect the peristaltic movements of various parts of the equine intestinal tract [2]. In the present study, there was no correlation between the wither height or body mass and the number of peristaltic movements.

An increased number of peristaltic movements in ponies and miniature horses may be caused by an increased field metabolic rate [42,43] and faster weight gain in these animals compared to large-breed horses fed the same diet (so called „easy keepers”) [44]. There were also differences in the number of peristaltic contractions between individual horses, which may be associated with the amount of ingesta and individual intensity of the gastrocolic reflex [26]. The differences among the studied horses may also be caused by the duration of the peristaltic movement observation. It may be worthwhile to examine the contractions for a longer period of time (e.g. three minutes) to determine if this would provide more precise results.

This study had several limitations. Primarily, it was carried out only on clinically healthy horses. In order to determine the reference range of the colonic localization, wall thickness and peristaltic motility in ponies and miniature horses, it is necessary to examine animals with pathological changes of the colon (for example wall thickening caused by inflammation or torsion). Two other limitations are lack of a full-thickness biopsy of the intestinal wall and no *post-mortem* evaluation of the appearance and thickness of the colonic wall [45]. All the horses included in the study were deemed healthy based on the history provided by the owners and by clinical examination and did not have any signs of digestive tract disease.

These results provide the first physiological ultrasound features of the RDC in ponies and miniature horses. The results confirm the presence of significant variability in the location, wall thickness and number of peristaltic contractions between small breed and large breed horses. These differences are important for professionals working with small breed horses. They should be taken into consideration in the diagnosis of intestinal disease. In addition, differences in the exact ultrasound location of the RDC in ponies and miniature horses may enhance a more precise FLASH protocol for miniature horses.
